# ERRATUM: Hypertension is the underlying cause of death assessed at the autopsy of individuals

**DOI:** 10.1590/1980-220X-REEUSP-2024-ER22en

**Published:** 2024-09-23

In the article “Hypertension is the underlying cause of death assessed at the autopsy of individuals”, with the DOI: http://doi.org/10.1590/S1980-220X2018006103457, published by the journal: “Revista da Escola de Enfermagem da USP, volume 53 of 2019, e03457, on page 8:

**Now read:**

## Financial support

This study was financed in part by the Coordenação de Aperfeiçoamento de Pessoal de Nível Superior – Brasil (CAPES) – Finance Code 001.

