# Supplementary material for: ERRATUM: Hypertension is the underlying cause of death assessed at the autopsy of individuals
Source: Rev Esc Enferm USP. 2024 Sep 23;58:e2024ER22. doi: 10.1590/1980-220X-REEUSP-2024-ER22en (PMC11421566; doi:10.1590/1980-220X-REEUSP-2024-ER22en)
Supplement: Translation

# Translation: ERRATA: A hipertensão arterial é causa subjacente de morte avaliada na autópsia de indivíduos

**DOI:** 10.1590/1980-220X-REEUSP-2024-ER22pt

No artigo “A hipertensão arterial é causa subjacente de morte avaliada na autópsia de indivíduos” com número de DOI: http://doi.org/10.1590/S1980-220X2018006103457, publicado na Revista da Escola de Enfermagem da USP, volume 53 de 2019, e03457, na página 8:

**Leia-se:**

## Apoio financeiro

O presente trabalho foi realizado com apoio da Coordenação e Aperfeiçoamento de Pessoal de Nível Superior – Brasil (CAPES) – Código de Financiamento 001.
